# Computed Tomography Analysis for Postoperative Muscle Changes in Patients With Ankylosed Hips Who Underwent Total Hip Arthroplasty: Three Case Reports

**DOI:** 10.7759/cureus.82654

**Published:** 2025-04-20

**Authors:** Shinya Tsujiku, Hyonmin Choe, Kazuma Miyatake, Hiroyuki Ike, Ken Kumagai, Naomi Kobayashi, Yutaka Inaba

**Affiliations:** 1 Department of Orthopaedic Surgery, Yokohama City University School of Medicine, Yokohama, JPN; 2 Department of Orthopaedic Surgery, Yokohama City University Medical Center, Yokohama, JPN

**Keywords:** activities of daily living, ankylosed hip, ct-based navigation, muscle volume, total hip arthroplasty

## Abstract

In patients with ankylosed hips, activities of daily living (ADL) are often restricted because of the limited range of hip motion and adjacent joint disorders that cause severe low back and knee pain. Total hip arthroplasty (THA) can improve hip mobility and reduce adjacent joint pain; however, muscle atrophy around the hip joint potentially interferes with ADL improvement. Herein, we describe the seven-year clinical outcomes of computed tomography (CT)-based navigation THA in three patients with ankylosed hips. Additionally, we report the longitudinal changes in muscle atrophy; the muscle volume around the hip joint was measured using CT analysis. The three patients include one woman and two men, aged 75, 65, and 73, respectively, who underwent THA using a CT-based navigation system. None of the patients experienced THA post-operative complications, and all were able to walk with a cane and sit freely in a chair. The hip function of the Harris Hip Score improved from a mean of 71.0 points to 86.6 points at seven years postoperatively. The volume of the gluteus maximus muscle increased in all patients (mean +10.1%); however, the gluteus medius and minimus decreased in all patients (mean, -42.2% and -51.0%). THA with CT-based navigation for ankylosed hips improves ADL, hip joint function, and muscle volume of the gluteus maximus in mid-term clinical results; however, muscle volume recovery in the gluteus medius and minimus can be insufficient.

## Introduction

An ankylosed hip is a condition in which the acetabular and femoral heads are osseously fused. Ankylosis may be caused by inflammatory arthritis that causes spontaneous fusion, surgical arthrodesis of the hip joint due to deformity, infections, or an unknown cause of coxitis [1-3]. Usually, patients with ankylosed hips do not suffer from hip pain; however, they suffer from restrictions in activities of daily living (ADL), such as sitting and lying down, because of restricted hip joint range or adjacent joint disorders that cause lumber pain and knee pain [4]. Although total hip arthroplasty (THA) potentially improves ADL in patients with ankylosis by increasing their range of motion, recovery of muscle strength around the hip joint is poor in the early postoperative stage owing to atrophic changes in the hip muscles from long-term disuse [5,6]. Therefore, THA for ankylosed hips poses the risk of high complication rates of postoperative hip dislocation and gait dysfunction due to muscle weakness in the postoperative period. Although recovery of these muscles is expected after THA in patients with ankylosed hips, changes in muscle volume and quality over time have not been previously investigated.

Here, we report the clinical outcomes of three cases of ankylosed hips seven years after THA using computed tomography (CT)-based navigation, and the quantification of muscle volume changes over time. 　

## Case presentation

**Figure 1:**
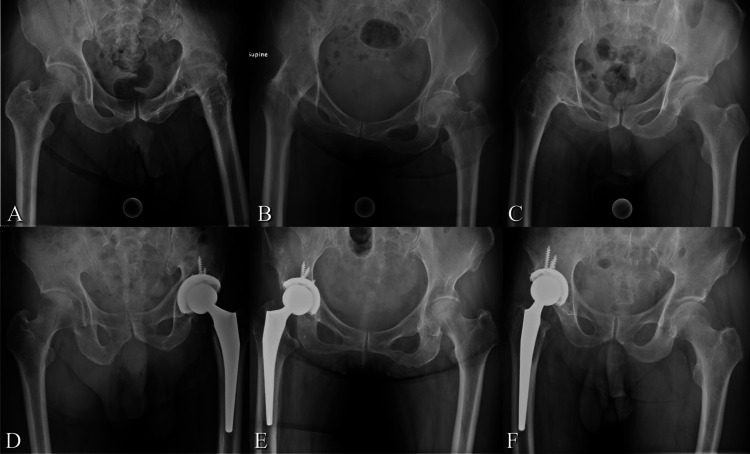
Perioperative hip joint radiographs. A, B, C: Preoperative hip radiographs of Cases 1, 2, and 3. D, E, F: Postoperative hip radiographs of Cases 1, 2, and 3. Case 1: A 65-year-old man; Case 2: A 73-year-old man; Case 3: A 75-year-old woman

**Figure 2:**
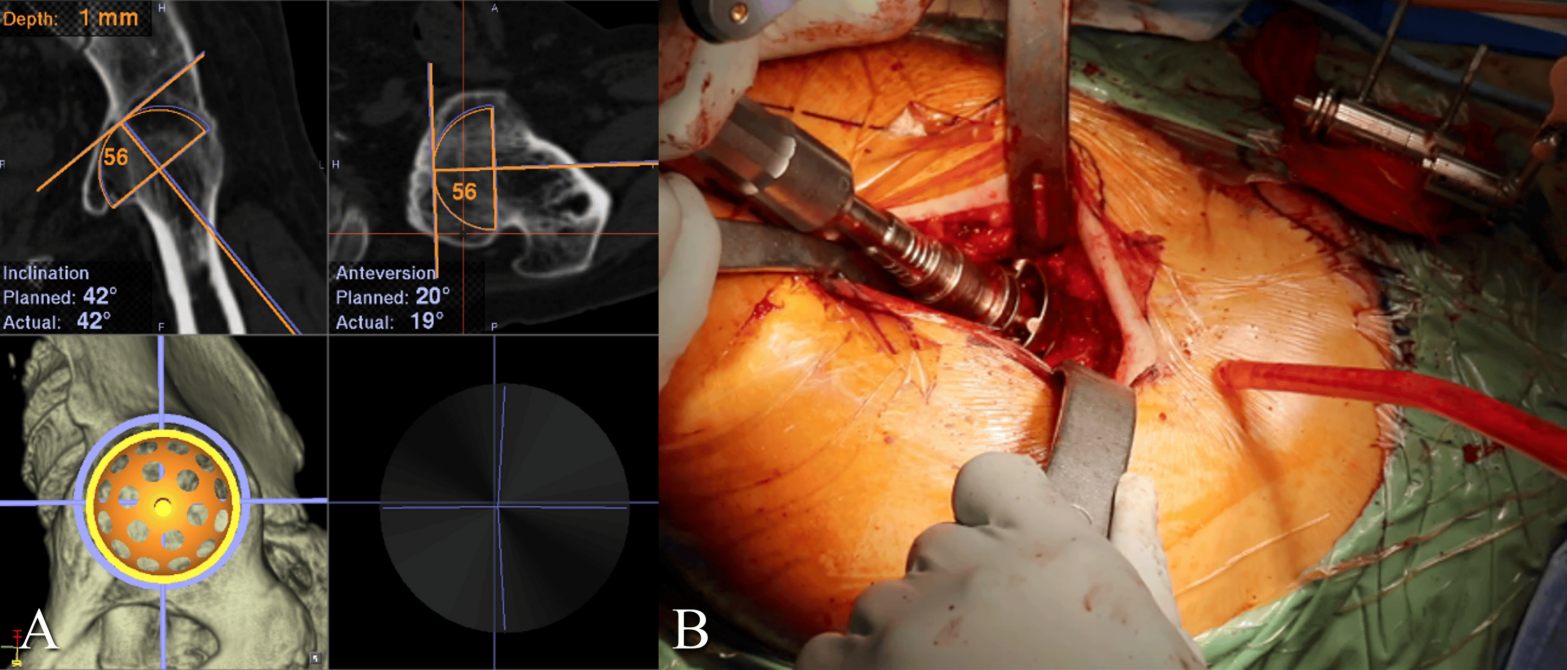
Computed tomography navigation images obtained during the operation for Case 1. A. The image of navigation during the reaming of acetabular reaming. B. The image during the reaming of acetabular reaming.

**Figure 3:**
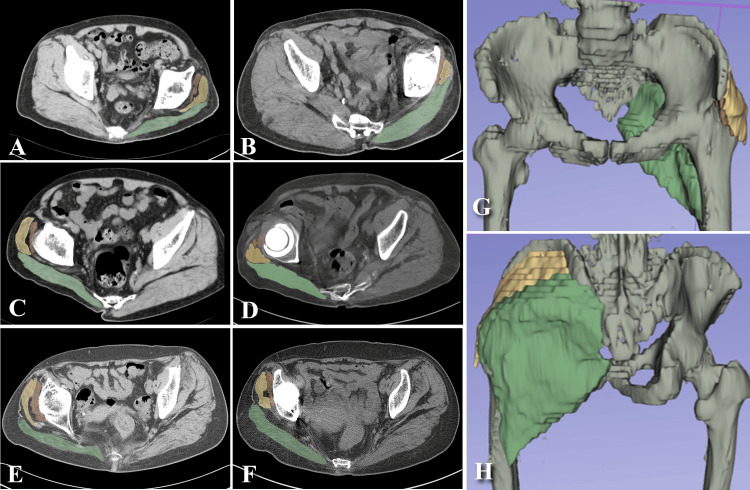
Measurement of muscle volume around the hip. A, B. Plane CT axial image of Case 1 before and seven years after operation. C, D. Plane CT axial image of Case 2 before and seven years after operation. E, F. Plane CT axial image of Case 3 before and seven years after operation. G, H. 3D images of Case 1 before operation By tracing the cross-sectional areas of the gluteus maximus (green), gluteus medius (yellow), and gluteus minimus (red) on every third slice of the CT images using 3D image analysis software (3D slicer), the volumes of the reconstructed muscles were quantified. Case 1: A 65-year-old man; Case 2: A 73-year-old man; Case 3: A 75-year-old woman

Three patients with ankylosed hips underwent THA using a CT-based navigation system at our hospital between July and September 2013. The patients comprised two men and one woman, aged 65 (Case 1), 73 (Case 2), and 75 years (Case 3), respectively. Tuberculous hip arthritis in two cases and infectious arthritis of unknown cause in one case caused the ankylosed hips. The ankylosed limb position was fixed at 0° flexion in two patients and at 45° in one. In all cases, CT was performed with a slice thickness of 1.5 mm from the first lumbar vertebra to the distal end of the femur before surgery, including the phantom (B-MAS 200; Kyoto Kagaku). THA was performed using a CT-based navigation system (Stryker, Mahwah, NJ, USA). The Trident PSL Cluster (Stryker) and Accolade TMZF (Stryker) were used for the cup and stem components, respectively (Figures 1-2). Cup inclination and anteversion were planned using a combined anteversion technique based on previous reports [7,8]. CT was performed before and three and seven years after the operation, and changes in the muscle volume of the gluteus maximus (Gmax), gluteus medius (Gmed), and gluteus minimus (Gmin) before and after the operation were evaluated. The measurement method was based on a previously demonstrated method [9], and each volume was measured using an open-source 3D slicer software (BWH, Cambridge, MA, USA) (Figure 3). To evaluate the clinical results before and after the operation, the Harris Hip Score (HHS) and hip joint range of motion (H-ROM) were measured.

Case 1 was a 65-year-old man who had no hip pain but suffered from limited ADL because of an ankylosed hip. He was referred to our hospital, aiming to regain the ability to sit in a chair. He was diagnosed with hip joint infection of tuberculosis and underwent the operation of hip fusion when he was five years old. He had a history of hypertension, hyperlipidemia, and cerebral infarction without paralysis. The radiograph of the pelvis shows an ankylosed hip on the left side. He underwent THA using a CT-based navigation system (Figure 1A, 1D). During the seven-year follow-up period, H-ROM and daily activity improved (Table 1). The preoperative HHS improved from 75 to 88 points. The patient could walk with a cane seven years postoperatively and sit freely in a chair owing to the improvement in H-ROM. The volume of the Gmax increased postoperatively, whereas those of the Gmed and Gmin decreased. The patient did not complain of low back or knee pain before or after the operation.

**Table 1:** Clinical outcomes of the three cases Pre-op, preoperative; Post-op, postoperative; HHS, Harris Hip Score; H-ROM, hip joint range of motion; Gmax, gluteus maximus; Gmed, gluteus medius; Gmin, gluteus minimus; VAS: visual analog scale

Variable		Case 1		Case 2		Case 3	
		Pre-op	7-year post-op	Pre-op	7-year post-op	Pre-Op	7-year post-op
HHS (points)		75	88	82	90	62	82
H-ROM (°)	Flexion (°)	0	110	45	80	0	90
	Abduction (°)	0	40	0	30	0	35
Muscle Volume (cm3)	Gmax	301.7	380.1 (126%)	351.1	378.6 (108%)	313.6	303.1 (97%)
	Gmed	101.2	59.7 (59%)	110.1	49.7 (45%)	96.4	66.7 (69%)
	Gmin	21.2	9.4 (44%)	27.2	16.9 (62%)	31.5	12.6 (40%)
Pain VAS	Low back	0	0	0	0	85	80
	Ipsilateral knee	0	0	0	0	60	90
	Contralateral knee	0	0	0	54	50	70

Case 2 was a 73-year-old man who had difficulty sitting and sometimes felt low back pain. He was diagnosed with hip joint infection when he was 10 years old and underwent conservative treatment, but the cause was unknown. He had a history of catheterisation for acute myocardial infarction and total knee arthroplasty for osteoarthritis of the ipsilateral knee. The radiograph of the pelvis shows an ankylosed hip on the right side. He underwent THA using a CT-based navigation system (Figure 1B, 1E). Patients experienced improvements in hip function and increased muscle volume in the Gmax. However, despite the improvement in hip function, the Gmed and Gmin showed postoperative reduction. This patient had no lower back pain but had postoperative contralateral knee pain, likely due to increased activity and contralateral loading.

Case 3 was a 75-year-old woman who suffered from limited activities of daily living and lower back and bilateral knee pain. She was diagnosed with hip joint infection of tuberculosis and underwent the operation of hip fusion when he was 32 years old. There is no other particular medical history. The radiograph of the pelvis shows an ankylosed hip on the right side. The THA was performed using a CT-based navigation system (Figure 1C, 1F). During the seven-year follow-up period, H-ROM and daily activity improved, while low back and knee pain persisted or worsened after the operation. Hip function of HHS improved; however, the muscle volume of Gmax remained, and Gmed and Gmin showed a reduction at seven years postoperatively.

## Discussion

In our study, we showed that THA for ankylosed hip joints leads to improvements in postoperative ADL. However, no improvement in muscle volume was observed in the atrophied Gmed and Gmin. This is probably due to long-term atrophic changes in Gmed and Gmin that limit their potential for hypertrophy even after THA. Notably, a reduction in muscle volume of these muscles was observed in all cases, even after seven years of rehabilitation. This finding represents critical information that surgeons must recognise and clearly explain to patients when considering THA for ankylosed hips. On the other hand, Gmax, which did not show extensive preoperative atrophy, showed improvement in muscle volume postoperatively. This suggests that for functional recovery, such as ADL and gait improvement, it is essential to instruct patients in compensatory muscle use strategies to substitute for the function of Gmed and Gmin.

Severe trauma or infection of the hip joint at a young age is a major cause of spontaneous ankylosis or surgical arthrodesis of the hip [10]. Although the ankylosed hip joint is generally painless, it causes significant ADL restrictions, such as difficulty in sitting and limitations in supine positioning. Long-term compensatory mechanisms for restricted hip motion often lead to degenerative pain in the lumbar spine, ipsilateral knee, and contralateral hip joint [1,11]. THA has been reported to relieve or eliminate pain in adjacent joints and to improve hip function and ADL [10, 12-13]. However, performing THA in ankylosed hips is technically demanding due to bone deformity, atrophy, and contracture of surrounding soft tissues, effects of previous surgery, and altered anatomical landmarks. Therefore, in our cases, THA was performed using a CT-based navigation system. This allowed for precise cup placement and enabled restoration of hip range of motion (ROM) without the heightened risk of dislocation. The regained ROM significantly contributed to improved hip function and ADL, particularly the ability to sit deeply or squat, resulting in a high level of patient satisfaction.

Nonetheless, some patients continued to experience low back pain and knee pain. These symptoms may be attributable to excessive biomechanical stress on the lumbar spine and knees as a result of compensating for hip instability. Although age-related degeneration over the seven-year postoperative period may also be a contributing factor, the persistence of pain in Case 3, who had preoperative low back and knee pain, suggests that THA in ankylosed hips may have limited effectiveness in alleviating pain in adjacent joints.

At seven years postoperatively, the mean hip ROM in our ankylosed hip cases was 93° in flexion and 35° in abduction, which is inferior to outcomes typically reported in THA for primary osteoarthritis [14,15]. Nevertheless, all patients reported satisfaction with the surgery. Thus, while THA for ankylosed hips can lead to substantial improvements in ADL, particularly enabling sitting and supine positions, it is essential to inform patients that muscle atrophy in the Gmed and Gmin may persist and that symptoms related to adjacent joint disorders may not fully resolve. Shared decision-making is crucial when considering this surgery.

## Conclusions

Accurate total hip arthroplasty using CT-based navigation for ankylosed hips improves ADL and hip joint function and enhances the muscle volume of the Gmax in the mid-term. However, recovery of muscle volume in Gmed and Gmin may remain insufficient, and these limitations should be taken into account when evaluating surgical outcomes.
